# Proximal Humerus Reconstruction after Tumor Resection: An Overview of Surgical Management

**DOI:** 10.1155/2021/5559377

**Published:** 2021-03-19

**Authors:** Antonio D'Arienzo, Edoardo Ipponi, Alfio Damiano Ruinato, Silvia De Franco, Simone Colangeli, Lorenzo Andreani, Rodolfo Capanna

**Affiliations:** Department of Orthopaedic and Trauma Surgery, University of Pisa, Pisa, Italy

## Abstract

Proximal humerus is one of the anatomical sites that are most frequently involved by bone and soft tissue malignant tumors. Alone or in association with adjuvant treatments, surgery represents the main therapeutic option to treat and eradicate these diseases. Once the first-line option, in the last decades, amputation lost its role as treatment of choice for the large majority of cases in favor of the modern limb sparing surgery that promises to preserve anatomy and—as much as possible—upper limb functionality. Currently, the main approaches used to replace proximal humerus after a wide resection in oncologic surgery can be summarized in biological reconstructions (allografts and autografts), prosthetic reconstructions (anatomic endoprostheses, total reverse shoulder prostheses), and graft-prosthetic composite reconstructions. The purpose of this overview is to present nowadays surgical options for proximal humerus reconstruction in oncological patients, with their respective advantages and disadvantages.

## 1. Introduction

Proximal humerus is the fourth most common localization for primary bone tumors, the first between the bones of the upper limb, representing the site of 10%–15% among all osteosarcomas and 10% among Ewing sarcomas [1, 2]. In addition, humerus is the second bone for metastatic involvement [3]. Shoulder as a whole is also the third anatomical site for incidence of soft tissue sarcomas in mankind, with deltoid muscle and supraspinatus fossa to be the most frequently involved sites [4, 5].

The combined incidence of bone and soft tissue sarcomas of the shoulder is about 0.25 per 100000 individuals [5, 6].

Surgical approach to tumors of the proximal humerus, just as much as the neoplasms involving other areas of the musculoskeletal system, requires preoperative work-up; shoulder function, neurological and vascular status, alongside with patient's general health must be accurately assessed.

If once amputations and arthrodesis were considered the approach of choice after massive resections, through the decades, the progress of modern surgery provided new and better opportunities to orthopaedic oncologists. Advances in oncology, imaging, and surgical technologies led the way to safer and more accurate resections, increasing the chances to achieve free-of-disease surgical margins and therefore making local recurrence a less frequent event in the postoperative phase. The reconstructive phase likewise saw the flourishing of several different approaches to restore—as much as possible—the preexisting shape and functionality of the shoulder. Osteoarticular allografts, bone autografts, prostheses, and graft-prothesis composites represent the current treatment of choice for proximal humerus reconstruction. All the aforementioned solutions guarantee the static repair of the shoulder in association with atleast partial restoration of its articularity. Overall, the objectives of oncological surgery for the treatment of proximal humerus neoplasms can be resumed in (a) removal of the tumoral mass with wide margins in order to reduce the risk of local recurrence, (b) limb sparing, and (c) restitution of the arm's length and shape in association with a shoulder mobility that could allow restitution of patient's activities of daily living, all making sure to maintain low the risk of local or systemic complications [7–18].

So far, consensus about the best reconstructive technique is far from being established [19–22]. The most suitable approach can vary depending on patients' age, clinical picture (considering comorbidities as well as life expectancy), functional demands, and the eventual sacrifice of rotators cuff, deltoid muscle, or axillary nerve through the procedure [22–28].

The aim of our study is to review the surgical management of tumors affecting the proximal humerus and evaluate the correct indications for every reconstructive option, in consideration of their advantages and disadvantages.

## 2. Options for Surgical Reconstruction

Upper limb sparing surgery often needs wide bone and soft tissue resections in order to achieve wide disease-free surgical margins. The tissue loss, if not treated properly, could impair both the shoulder and the arm from a static and a dynamic point of view. Arm's length and shape, as well as the articular ROM (range of motion) of the shoulder, are crucial for the functionality of the upper limb, which in turn is pivotal to restore patients' autonomy and therefore increase their postoperative quality of life.

According to the most recent advances in orthopaedic oncology, reconstruction of the proximal humerus can be performed with—among the others—autografts, allografts, implanted prostheses, or prosthetic-biological composites [13, 18, 23, 24, 28–35].

Despite the increasing interest on the topic and the spread of reports in literature, the choice between the various approaches to reconstruct proximal humerus remains controversial, with studies that come down for or against the various options available at the moment [9, 16, 35–37].

In the following sections, we are going to expose different reconstructive techniques, evaluating their pros and cons in association with their functional results and complications rate, all to offer the readers a state-of-the-art overview about the modern surgical opportunities to recreate functional proximal humerus after wide resection.

### 2.1. Graft Reconstructions

Homologous and heterologous transplants of bone tissue as a concept have its roots at the dawn of civilization. In the Old Testament, Adam's rib is used to create Eve, while in the Greek mythology, Haephestus manufactures an ivory shoulder for Tantalus' son, Pelops [38]. However, it was not until the 1970s, with the evolution in cooling techniques and a detailed comprehension of the pivotal role played by vascularization that allografts and autografts established themselves as reliable reconstructive options in orthopaedic surgery.

To this date, the use of both allografts and autografts as reconstructive options after the resection of proximal humerus has been largely described in literature. Although the results are often encouraging, it is important to point their advantages and the disadvantages, in order to make sure they represent the best therapeutic option for the single patient.

#### 2.1.1. Allografts

In particular, young and active patients with few comorbidities are the most suited to be treated with an allograft; their long life expectancy, in association with good healing capacities, justify the use of bone grafts considering the high chances of osteointegration as well as the possibility to provide them a life-long restoration of the shoulder [39]. Unlike prosthesis, grafts do not have default duration, and if the treatment is successful, revision surgery might not be necessary even after decades. Mourikis et al. in 2007 had an implant survival rate of 67% 16 years after surgery [40]. Aponte-Tinao et al. in 2013 reported a rate of 65% after 10 years [32].

Graft functionality often gets better as the years go by, and osteointegration is perfected, as opposed to prosthetic reconstructions that undergo unavoidable wear after many years of use. Prosthesis are also burdened by the risk of dislocation [13, 30, 41–47], aseptic loosening [29, 48, 49], and infection [48], a threat that in grafts is partially mitigated by the presence in loco of the patient's immune system and the absence of large metal segments ideal for the deposit of bacteria's biofilm [50].

Another intraoperative advantage in favor of allografts is attributable to the fact that modern cryoconservation technologies allow maintaining viable articular chondrocytes, so that the graft articular surface may include a living chondral surface for the reconstructed shoulder joint, providing a lower risk of postoperative osteoarthrosis [9, 10]. Finally, grafts can ensure sites for the reattachment of rotator cuff, deltoid, and pectoralis muscles respecting patient's own anatomy in a fashion that could never be performed with prosthesis [26]. For these reasons, in many cases, allograft reconstruction is a valid option and can represent the first choice of treatment [23, 28, 30, 40, 51–55].

On the other hand, allografts are not exempt from defects. Their use presupposes a rapid and successful colonization of atleast a part of the bone scaffold by the living cells of the receiving patient; in case it does not happen, the whole procedure would be destined to failure. Such an eventuality is more likely to happen in patients with advanced age, metabolic, or neurovascular disorders (such as type 2 diabetes); all factors negatively affect patient's healing capacity. In oncologic patients such as the ones subject of our discussion, pre and postoperative treatment with local radiotherapy or even systemic chemotherapy may also represent an obstacle to a proper osteointegration [38, 39]. Inhibition and death of the cells that surround the graft, alongside with alterations of the local homeostatic balance, make the allograft colonization harder to achieve and even more complicated, in case of postoperative irradiation which would make the implanted bone scaffold even less suitable for eventual settlers. Another concern associated with the use of allografts is their relatively high risk of postoperative complications. Unfortunately, the use of osteoarticular allografts has been often associated with high rates of local complications such as chondrolysis, subchondral collapse, and implant fractures [9, 13, 18, 23, 24, 28–35, 40, 51, 54, 55]. These adverse events often require revision surgery, leading to a significant decrease in terms of implant [30]. Aponte-Tinao et al. [32], albeit praising the good overall results of this technique, described among their shoulder graft reconstructions a risk of failure that amounted to 23%. Comparable rates were shown also by DeGroot et al. (22%) [51], Jamshidi et al. (22%) [52], and Ogink et al. (23%) [53]; others had even higher rates, such as Mourikis et al. (33%) [40], Yao et al. (38%) [54], or Potter et al. (29%) [30]. Potter et al. pointed an overall success lower than the ones achieved by the same authors using other reconstructive approaches. In fact, allografts, compared to endoprostheses and allograft-prosthetic composites, had more complications and required almost twice as much reinterventions. Potter, as well as van de Sande et al. [23], therefore preferred other methods to allografts precisely as a consequence of their lower complication rate. Other studies such as the one by Rödl et al. [28], for their part, showed complication rates remarkably lower (Table 1).

Implant fractures are among the most feared major complications that might occur after graft surgery (8–53%), being one of the main reasons for its failures [23, 28, 30, 40, 54]. In their population, Bus et al. [56] found that proximal humerus implants in particular had a significantly higher incidence of fractures compared to other anatomical districts. Literature suggests that the addition of cement inside the graft's intramedullary canal might be a strategy to prevent this eventuality. DeGroot et al. [51] and Jamshidi et al. [52] indicated how cement-augmented allografts seem to be associated with a lower rate of diaphysal and metaphysal fractures in comparison to nonaugmented allografts. This strategy could theoretically be an effective and inexpensive solution to decrease both the risk of fractures and infections, although operators should always keep in mind the risks associated with the use of PMMA [57].

Literature reports postoperative functional (MSTS) scores that range between 21.3 and 25.5 as represented in Table 1. Rödl et al. [28] obtained a mean MSTS score of 22.2, a solid value, but nevertheless lower than the ones boasted by prostheses (23.7) and autografts (24.6). Potter et al. [30] treated 17 patients with osteoarticular allografts getting a mean MSTS score of 21.3, a result that was slightly better than the one of endoprostheses (20.7) but worse than the APC's group (23.7).

#### 2.1.2. Autografts

Autologous bone grafting involves bone segments obtained from the patient himself in donor sites that can be near or far from the recipient one. Bone tissue can be picked up from nonessential bones, whose sacrifice would not cause major repercussions on patient's quality of life. Among these, not only fibula is most frequently used and described in literature but also other donor sites can be used for the purpose, such as ribs or the neighboring scapula and clavicle [14, 27–29, 58–70].

Harvested bones require continuous blood supply to survive in the receiving area. Their vascularization must thus be preserved, if the donor site is adjacent to the shoulder, or restored anastomosing the vascular peduncle of the graft with local vessels, if the tissue is taken from a far region.

In comparison to allografts, autografts do not only serve as a scaffold that receives the nearby osteoblasts (osteoconduction) but also have a greater tendency to stimulate the osteoprogenitor cells of the nearby tissues to differentiate into osteoblasts and begin new bone formation (osteoinduction).

Moreover, consisting in vital and metabolically active bone tissue, autologous grafts bring their own osteogenesis potential to the receiving site, giving their contribution to the local bone growth and postoperative healing. This is supposed to translate in better fusion rates, mechanical strength, and infection resistance.

These factors theoretically mean that allografts have the best osteointegrative potential among the other reconstructive approaches available today and may represent the treatment of choice especially for young patients with long life expectancy [71–75]. Patients still in the process of growing, in particular, could benefit from the inclusion of an open growth plate inside the implant, allowing graft's growth in length and miming the structural evolution of the native bone in the following years [76–78]. Another positive factor is the fact that, since the donor and the receiving patient are the same individual, there is no risk of graft-versus-host-disease.

At the same time, however, this approach is not free of disadvantages. The stock of bones suitable to replace proximal humerus in terms of size and shape is limited, and their removal always deprives the donor site of one of its natural elements [78]. In practice, in order to harvest, an allograft is necessary to extend the surgical access (if the graft is taken close to the receiving site) or even to create a second access centered on the bone of choice; in either case, the result is lengthening of surgical time, with consequential increasement in blood loss and higher complications risk also due to anesthetic procedures [28]. Unlike allografts, it is also hard to obtain a vascularized bone graft provided with an articular surface covered with living chondral tissue, which would represent an advantage to restore properly the distal side of shoulder articulation. For these reasons, autografts are at times considered unattractive to reconstruct proximal humerus after major resections.

We report a summary of the autograft techniques mostly described in literature as follows.

**Figure 1:**
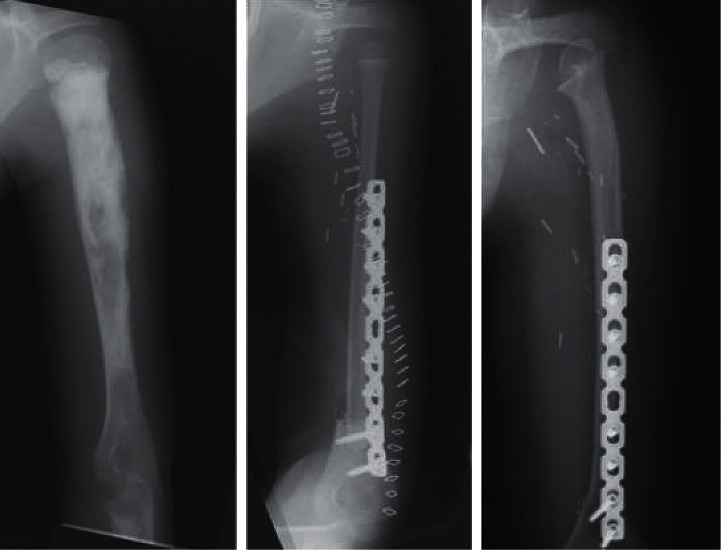
A FVFG (free vascularized fibular graft) is used to replace proximal humerus in a young growing patient who underwent massive resection to treat a malignant bone tumor. The proximal growing plate of the fibula allowed the autograft to increase its length, mimicking the growth of the native proximal humerus.

*(1) Free Vascularized Fibula Graft (FVFG)*. Fibula is one of the most suitable donor bones to replace proximal humerus. Free vascularized fibular graft (FVFG) was first described in 1975 by Taylor et al. [79] and since that moment emerged as a reliable option for a large variety of reconstructions, including large defects of the upper and lower extremities. Its popularity can be attributed to the fibula's favorable anatomy, being long and straight, providing up to 26 cm of bone and having a dependable dual endosteal and periosteal blood supply [80]. These features make the FVFG particularly suitable to replace long bones such as humerus, even after a wide resection. Relatively low donor site morbidity was another point of interest [58, 81]. In pediatric patients, surgeons may include proximal fibula and its growing plate inside the implant, so that the graft conserves its growing potential and will allow the lengthening of the recreated humerus in the future [64] (Figure 1). Although the large variety of patients does not show signs of local complications in the donor site, particular attention should be given to the postoperative knee stability. To avoid this issue and increase articular firmness, we recommend fixing the detached lateral collateral ligament (LCL) and biceps tendons to the lateral surface of the tibial plateau with suture anchors.

In 1999, Wada et al. [14] used a free vascularized fibular graft as a functional spacer to replace proximal humerus, practicing a sling procedure to preserve passive scapulohumeral mobility. 8 patients with a mean age of 27 years (10–47) were treated using the contralateral or ipsilateral fibula whose proximal pole was fixed to the proximal trump of the humerus with a dynamic compression plate. Fibular head was suspended and connected to the remaining part of the scapula by the tendons of the tendon grafts and transient stainless steel wires. In some cases, a latissimus dorsi flap was required to create adequate muscle coverage. The population treated had good functional outcomes (MSTS 23.7, 79%), and no functional problem was observed eventhough 5 of the 8 patients had radiographical signs of absorption, collapse, or fracture of the fibular head. Manfrini et al. [64] and Bilgin [63] obtained comparable results, respectively, in 2011 and 2012 (Table 2).

FVFG can also be used as a bone substrate to perform arthrodesis [61, 62]. In orthopaedic oncology, shoulder arthrodesis (with fibular graft alone or associated with allografts) is currently indicated for patients with brachial plexus injury, rotators cuff, and deltoid muscle dysfunction or in case of failed prosthetic arthroplasty [81]. This approach was widely accepted as a salvage procedure, especially if the resection of the proximal humerus was larger than 6 cm [63]. Surgical procedure may involve the use of one [82] or two plates, with this latter addition that is supposed to improve fixation strength and reliability [83, 84]. Wang et al. [33] reported that patients with extensive proximal humeral bone loss and abductor muscle sacrifice treated by arthrodesis had superior function and strength compared with others who had prosthesis or allograft-prosthesis composites (APCs). A further advantage of the autografts, in comparison to prosthetic reconstructions, is due to the progressive degradation these later suffer over the time, whereas biologic reconstructions strengthen over the years as bone hypertrophy occurs [63].

Bilgin [63] and Fuchs et al. [85] reported good functional outcomes (MSTS scores, respectively, 23 and 22.7) despite the severe loss of shoulder articularity. Unfortunately, these solid results were atleast partially overshadowed by a high risk of postoperative complications that often made necessary a revision surgery. In 2005, Fuchs et al. [85] had a failure rate of 43%, mainly attributable to nonunions, fractures, or infections. Slightly better results were obtained by Bilgin in 2012 [63] who used double-barrel grafts in some patients to reduce their risk of postoperative fracture. In summary, reconstructions that involve free vascularized fibular grafts (FVFGs) can restore the humeral length and provide active bone healing, at the price of a complex long-lasting surgical procedure and a relatively high risk of postoperative complications.

*(2) Scapular Pillar Graft.* Scapular pillar bone graft was first described by Gilbert and Teot in 1981 [86]. Their innovative study led the way to its wider use in the following decades for the treatment of humeral bone losses, especially in young tumoral patients with long life expectancy and medium-to-high functional requests [67, 87–89]. The technique, presented in detail by Le Reun et al. in 2019 [89], consists in the use of the lateral border of the scapula as an autologous vascularized bone graft to bridge the defect resulting from the humerus resection. Scapular pillar must be resected involving also the inferior portion of the glenoid, being careful to preserve the circumflex scapular vessels that supply blood to the bone segment of interest. Once the graft has been mobilized, the operator should rotate it laterally, using the inferior pole of the glenoid as a pivot. With a useful width of at least 2 cm and a length up to 15 cm, this vascularized graft can be solidarized to the distal humerus and to the remaining scapula performing an arthrodesis. The base of the scapular pillar itself is the best anchoring point for the scapular end [89, 90]. Compared to most frequently described vascularized fibular graft, the use of scapular pillar allows a faster procedure and does not require a second surgical field nor microsurgical anastomoses, avoiding complications such as vascular occlusions. On the other hand, the major limitation to its use might be the length of the graft that makes this surgery compatible only with resections shorter than 15 cm, according to Le Reun et al. [89] and Amin and Ebeid [66]. Therefore, this technique is primarily used to replace small defects, even though Padiolleau et al. [67] proposed the combination with free autologous grafts from other body sites (the iliac crest, fibula, tibia, or coronoid process) to increase reconstruction's length and stability. Moreover, this approach is contraindicated for cases with large metastasis of kidney cancer, since they might require preoperative embolization and consequentially could reduce the feasibility of local vascularized grafts. In terms of functional outcomes, Amin and Ebeid [66] testifies a mean MSTS score of 22.5 (75%) in their population of 16 patients, and Padiolleau et al. [67] had a mean value of 21.3 (71%) out of their 12 cases (Table 2). These results are comparable with the ones obtained using other procedures, and no major complications were found in either study.

*(3) Clavicula Pro Humero (CPH)*. Described for the first time by Sulamaa in 1963 to treat patients suffering of phocomelia, this technique was imported for the use in cancer patients at the beginning of the 1990s with the work of Sulamaa and Winkelmann [11, 91]. Surgery is performed cutting the clavicle at its medial third and returning it through a lateral pivot point represented by the acromioclavicular joint. Once verticalized, the graft can be synthetized to what remains of the native humerus directly or through an interposed graft in case the length of clavicula alone would not be enough to fulfill the bone gap. This technique does not require a second operative field nor microsurgical skills as the native blood supply is maintained through the thoracoacromial trunk. Clavicula pro humero allows the maintenance of upper limb length and good restoration of shoulder stability and mobility. Clavicle graft also provides reconstruction with growth potential due to the presence of the lateral growth plate of the clavicle, eventhough the lengthening could not match one of the contralateral humeri. For these features, this reconstructive approach can be considered a reasonable option in pediatric patients [68, 69]. On the downside, this procedure is complex and time consuming compared to allograft surgery. Kitagawa et al. showed that patients treated with CPH had a higher revision rate compared to the ones treated with prosthetic arthroplasty or osteoarticular allograft arthroplasty [27]. The main failure reasons in studies were graft fractures and infections. Rödl et al. in 2002 reported relatively good functional outcomes (mean MSTS score 23, 77%) but still with a high rate of postoperative complications and failures [28] (Table 2). Weighting in the balance advantages and disadvantages, clavicula pro humero can be viewed as an alternative in patients who cannot undergo prosthesis reconstructions because of their bone size and age or in countries where tumor prostheses are not available.

## 3. Prosthetic Reconstructions

Transversal studies testified that prosthetic reconstructions, compared to biological reconstructions, had fewer complications and a higher overall implant survival without this negatively influencing the postoperative functional outcomes [51, 92].

Advantages of prostheses are not only the relatively low complication rates but also the quick recovery of articular stability and upper limb movement [28, 36]. Newest designs brought further improvements in implants' complications-free survivorship [93] and fewer implant-associated complications [28, 47, 94, 95].

### 3.1. Anatomical Endoprostheses

Modern days' humeral endoprostheses sink their roots in the surgical and technical advances brought by Charles Sumner Neer II between the 1950s and the 70s. According to Neer, shoulder hemiarthroplasty was intended to ease local pain, preserve the normal anatomy of the site, and meanwhile provide sufficient functionality to the involved upper limb [96].

Following this line of fought, modern humeral megaprostheses can be substantially considered as articular spacers whose purposes are to ensure the respect of shoulder's anatomical overview and give back an articular mobility that, with the help of the elbow and wrist, could allow patients to carry out basic activities, such as bring the fork to their mouth, brush their teeth, or touch their hair [1, 28, 36, 47, 97–99].

**Figure 2:**
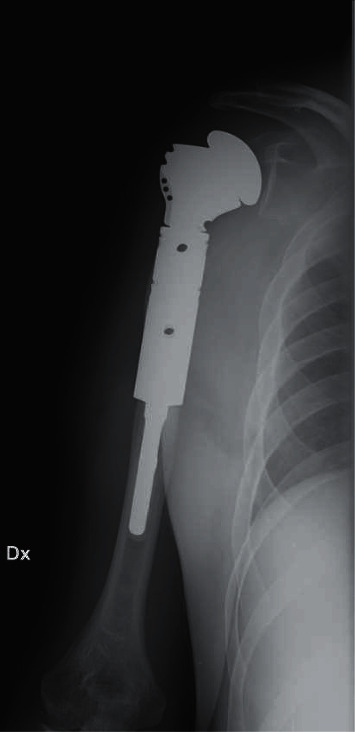
Anatomic megaprosthesis of proximal humerus implanted in our institution after massive bone resection due to resection of a malignant bone tumor.

Nowadays, many endoprosthetic systems are available on the market, and the most varied implants have been described in literature from the modular to the custom-made devices, with both cemented or uncemented fixation concepts (Figure 2) [13, 36, 41, 94, 100, 101].

From a functional point of view, the mean MSTS scores reported in literature ranged between 18 and 25 out of 30 (Table 3). Though the 25 reported by Angelini et al. [43] in 2017 was encouraging, the evaluation of endoprostheses' functional results remains controversial [47, 100]. Although these implants represent a good platform for the functionality of the elbow and the hand below, their stability is not always perfect and shoulder's active range of motion is limited, with above shoulder activities that are often precluded [41, 43, 61, 107–112]. These limitations reflect the loss of the rotators cuff and almost total impossibility to properly reattach tendons and articular capsule to nonbiological prosthesis. In order to alleviate these criticalities, oncologic surgeons proposed alternative solutions to increase articular stability. Asavamongkolkul et al. [13] covered their custom-made endoprostheses with Dacron aortic grafts (DACs) that were sutured to the labrum and secured around the endoprostheses to make shoulders more stable and prevent subluxations or dislocations. van de Sande et al. [23] and Raiss et al. [41] described the use of trevira tubes to cover prostheses and provide reattach sites to the surrounding soft tissues. The tube was fixed on the interior to the prosthesis and outside with the remaining parts of the capsule, tendons, and muscles to better link the implant to the nearby structures.

Due to their mediocre functional outcomes, articular shoulder endoprostheses are not to be considered among the most suitable approaches for young high-demanding patients. On the contrary, these reconstructions represent reliable options for patients of older age, with lower functional requests, and/or suffering of systemic diseases. For cases with diabetes, high infective risks, or other systemic healing deficiencies, for whom grafts might represent a gamble due to their high risk of implant failure, prostheses pose as reliable solutions. Metastatic patients, for example, could benefit from the implant of an endoprosthesis since it could give early unrestricted motion of the elbow, wrist, and hand, quickly eases the pain, and limits postoperative immobilization [102]. In addition, surgical times are relatively short (which implies lesser blood loss and systemic stress), and the implant longevity should not represent a problem, since it is likely to exceed patient's life expectancy [30].

As mentioned before, the risk of complications is relatively low, but not even this technique is completely free of risks. Mechanical issues and even failures are possible; eventhough ruptures are extremely rare, dislocation and subluxation are described as the main complications of megaprostheses, involving up to 20% of the cases in literature [23, 41, 47, 94, 95, 104].

Infections remain another serious concern, with a reported risk that varies widely, ranging from 2% to 40% [13, 93, 97, 113–123]. These eventualities, for how much uncommon, are factors surgeons should focus on before deciding for a prosthetic reconstruction.

### 3.2. Reverse Total Shoulder Prostheses

**Figure 3:**
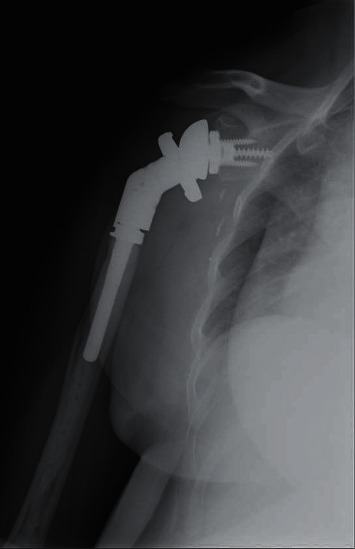
Reverse shoulder megaprosthesis implanted in our institution after massive bone resection due to resection of a malignant bone tumor.

Reverse total prostheses are implants in which the articular components switch; the socket is placed in the proximal humerus and the prosthetic ball is set on the glenoid (Figure 3). This design has been conceived to improve articular strength, stability, and range of motion, meanwhile keeping low the risks of dislocation and aseptic loosening.

For oncologic patients with proximal humerus resections, especially for those who lost their rotator cuff but maintained deltoid insertion and axillary nerve, reverse shoulder arthroplasty represents a reliable treatment that can generally lead to better functional results compared to hemiendoprosthesis. Mean MSTS scores described in literature ranged between 18 and 25.7 out of 30, with results that are on average higher than the ones obtained with endoprostheses. In comparison to these later, reverse total shoulder prostheses also provide better flexion and abduction. In fact, the functionality allowed by reverse shoulder prostheses does not limit to morphological support to the elbow and wrist but extents to active shoulder flexion and abduction generally above the 90 degrees, greatly increasing patients' possibilities in terms of daily living activities [17, 44, 46, 104, 124]. As described by De Wilde et al. in 2005, patients with proximal humerus and rotators cuff resection treated with reverse shoulder arthroplasty rely on the deltoid muscle to generate active shoulder function; therefore, surgeons must focus on optimizing the deltoid moment arm and muscular elongation to get better clinical results [17]. Boileau et al. also testified promising functional results in nononcological patients when reverse shoulder arthroplasty was used in combination with a modified latissimus dorsi and teres major tendon transfer. Cases showed an improvement of mean active elevation and external rotation range that could encourage a similar use in oncological field [125]. Another possible variation capable of increasing shoulder stability is the addition of synthetic tubes to surround the reverse prostheses, similar to what above described in the prior subchapter [23, 41, 46, 47, 126].

Reverse shoulder total prostheses should be performed mainly in case both deltoid and axillary nerves are preserved, in order to maximize the aforementioned good outcomes in terms of postoperative functionality.

The overall complication rate in literature amounted between 0 and 40%. The most common were scapular notching (mean risk between 0 and 30%) [44, 46, 104, 106], articular instability or dislocations (0–30%) [44–46, 49, 104], and aseptic loosening (0–13.3%) [49, 106]. Infective risk was low, since no study reported infection rates higher than 6.7% [45, 47, 49]. Depending on the study, the total failure risk was between 0% and 36% (Table 3). These percentages elect reversed shoulder arthroplasty as a relatively safe option for patients whose tumor and consequential resection damaged the rotators cuff but saved deltoid muscle and axillary nerve. Before choosing this surgical approach, anyway, surgeons should consider that prostheses for their own nature have limited durability. Eventhough modern conception devices can remain functional for decades, still they are not everlasting and their mechanical properties might not stand the test of time as well as grafts could. Young patients with long life expectancy could possibly run into of prosthesis decay; therefore, revision surgery would be necessary. On the contrary, device durability should represent nothing but a minor concern for midlife patients or cases with limited life expectancy.

## 4. Allograft-Prosthesis Composite (APC) Reconstructions

In orthopaedic oncology, surgical treatment of proximal humerus often leads to wide resections of both bones and soft tissues. Removal of joint capsule, rotator cuff muscles, or deltoid could be necessary in order to achieve a radical excision of neoplastic tissue. This could compromise shoulder stability after reconstruction, especially because of the intrinsic instability of the joint without the stabilizers complex [34].

Endoprosthesis and allograft, as described in previous chapters, are two of the most dominant reconstructive options for proximal humerus to this date. However, in the postoperative medium-term follow-up, both these techniques count several major complications that make revision surgery inevitable. Endoprostheses cannot always provide a valid reattachment for rotator cuff tendons, threating joint stability and ending in an increased risk of dislocation and subluxation.

Compared to endoprostheses, allografts have a number of advantages: restoration of bone stock and soft tissues attachment guarantee better stability, wider shoulder range of motion, and consequently improved functional outcomes. Nevertheless, reconstruction with allografts as well leads to major complications such as bone resorption, fracture of the graft, and nonunion at bone-graft interface frequently described in literature [127].

Humeral reconstruction can also be performed matching biological and prosthetic approaches that meet allograft-prosthesis composite reconstructions (APC). This method was first described in 1991 by Gitelis to overcome the considerable risk of allografts alone to suffer fractures and implant failures. The association of allografts with prostheses theoretically allows matching the structural advantages from both reconstructions. Allograft's possibility to anchor surrounding soft tissues and therefore increase articular stability is combined with prostheses' durability; a more rigid construct is created and lower risk of fractures, subchondral collapses, and dislocations are achieved [128].

Almost all the testimonies of APCs in the oncologic field are case series of patients who underwent intraarticular resections 1A or 1B according to the Malawer classification.

After an intraarticular 1A resection of proximal humerus, an allograft-reverse prosthesis composite should be taken into consideration, remembering that this reconstruction requires good bone stock on the glenoid, integrity of the axillary nerve, and preserved deltoid function to guarantee satisfactory functional outcomes [128]. Surgical procedure assumes the use of an appositely prepared frozen allograft to replace proximal humerus and accommodate the prosthetic stem; the proximal part of the prosthesis is cemented to the graft and the distal stem is inserted in the native bone canal, either cemented or press-fitted. Cementation of the distal stem end is recommended especially in elderly people with low bone quality; according to Lozano-Calderón and Chen [128], cement augmentation of the proximal stem in the graft increases rigidity of the construct and reduces the risk of allograft fracture [128]. Stem could be press-fitted in younger patients to compound osteointegration of the stem in the host bone and at the bone-graft interface. Moreover, uncemented stems could reduce the risk of implant aseptic loosening in a long-term scenario. The employment of a long prosthetic stem provides stability to the graft-bone interface, which could be additionally increased with fixation plates. At the end of bone reconstruction, correct reattachment of soft tissues to the graft must be made; in particular, capsule repair along with reinsertion of rotator cuff and deltoid tendon are essential for the recovery of shoulder functionality [34, 128, 129].

Abdeen and Healey [34] presented the largest series of patients undergone intraarticular or extraarticular resection, with 36 cases and a mean follow-up of 5 years. They underlined the importance of deltoid preservation to restore a good range of motion: patients in whom a partial or complete deltoid resection had been necessary, the sacrifice translated in unsatisfying abduction and forward flexion. However, this aspect did not significantly influence the MSTS score in the medium-term follow-up that has been lowered in the extraarticular resection instead [34].

In literature, medium-term results were generally satisfactory in terms of pain, shoulder range of motion, and quality of life, if compared with preoperative ones. Average MSTS scores, when calculated, were always higher than 18 (60%) with good outcomes on patients' emotional and acceptance points of view (Table 4).

As always, in orthopaedic practice, a surgical technique must be evaluated, weighing in the balance its functional results together with the risks it poses.

The most frequent complication detected by authors was delayed union or nonunion at the graft-bone interface. Nonunion rate does not seems to be significantly conditioned by the choice of cementation or press-fitting of the prosthetic stem into the host bone and even the employment of fixation plates to increase composite stability and rotation control cannot entirely prevent nonunions. El Beaino et al. [130] collected a series of 21 cases with a median follow-up of 97 months. In seven patients, a plate at the junction was positioned; three of them (43%) had delayed union. In parallel, the same complication occurred in 7 of the 14 patients (50%) without plates at the junction. All patients had received primary grafting at the interface. Black et al. [25] reported six cases of allograft-endoprosthesis composite, all treated with exclusively grafting at the interface, without plating. Only one of six developed nonunion at the junction that required surgical revision with new grafting and stabilisation with a plate. Nonunions were the main cause of implant revision, generally requiring new grafting at the allograft-host bone junction and sometimes the application of additional plates. Resolution of nonunion was always achieved after surgery, as testified by postoperative radiographical and clinical evidences of bone healing, with adequate bone callus and pain relief.

Also, shoulder dislocations or subluxations were not infrequent, similar to what was observed for prosthetic reconstructions alone. El Beaino et al. [130] described 12 cases of shoulder subdislocations, although revision surgery was necessary in one patient only.

Aseptic loosening, infection, and fracture rates were low, but when they occurred, revision surgery was always required.

No cases of resorption of the graft have been described in literature to this date; this might establish APCs as a suitable solution to overcome one of the biggest problems associated with the use of allografts alone [127].

All in all, proximal humerus reconstruction with allograft prosthesis composite is a valid option of treatment in oncologic patients that required wide bone resections. Their overall good functional results, associated with a better implant longevity compared to allografts alone, made the allograft-prosthetic composites (APCs), a relevant technique especially for younger patients with primary bone tumors and absence of systemic involvement [30]. Surgery is longer and more complex compared to allograft or prosthesis placement, and unfortunately, APCs are not lacking in complications neither. Nevertheless, they present advantages of both prosthetic and biological reconstructive approaches and can lead to a reduction of the classic complications that come along when using one of the two other techniques alone.

## 5. Conclusion

The choice of the most appropriate surgical treatment should result from an accurate overview covering not only resection extent but also patient' systemic and local clinical picture, life expectancy, and functional requests.

**Figure 4:**
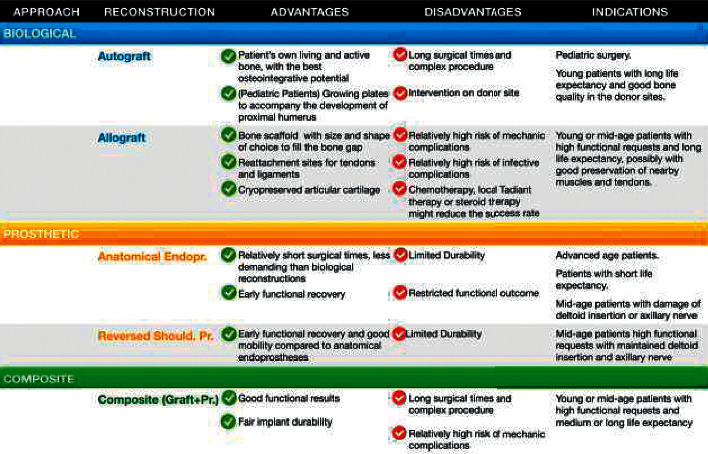
Schematic summary of the main advantages and disadvantages, with consequential indications, for the main reconstructive approaches to proximal humerus in oncologic surgery.

Although a unique line of thinking is yet to be established, modern literature suggests that biologic reconstructions are indicated mainly for young patients with long life expectancy and high functional requests. In fact, the osteointegration and mechanic performances promised by grafts could lead to great functional results through the long life of the implant. On the other hand, prosthetic reconstructions represent a reliable option especially for patients with medium or short life expectancy who could be allowed anyway to maintain decent upper limb functionality with a reasonable recovery time. A summary of advantages and disadvantages for each reconstructive strategy and the suggested indications in light of the aforementioned literature evidences and our own experience is shown in Figure 4.

Modern surgery provides a large variety of reconstructive solutions for proximal humerus after tumor resection, each one with its advantages and disadvantages; it is up to the surgeon to consider what patient needs and choose the technique that better suits the individual.

## Figures and Tables

**Table 1:** Summary data for allograft studies.

Study	Resection (cm)	Reconstruction	*N*	Age (y)	F-U (m)	Hardware issues	Graft resorptions	Fractures	Infections	PSA	Failures	MSTS score

DeGroot et al. [51]	12	Osteoarticular allograft	32	30	64	3% (1)	0	37% (11)	1	16% (5)	22% (7)	22.4
Jamshidi et al. [52]	15	Osteoarticular allograft	32	27	46	0	11% (4)	8% (3)	8% (3)	6% (2)	22% (8)	25.5
Mourikis et al. [40]	—	Osteoarticular allograft	32	42^*∗*^	192^*∗*^	0	0	19% (6)	13% (4)	16% (5)	33% (10)	—
Ogink et al. [53]	14	Osteoarticular allograft	32	47	—	6% (3)	2% (1)	28% (13)	4% (2)	13% (6)	23% (11)	—
Potter et al. [30]	15	Osteoarticular allograft	32	36	24	0%	0%	53% (9)	12% (2)	6% (1)	29% (5)	21.3
Rödl et al. [28]	16	Osteoarticular allograft	32	20	59^*∗*^	0	0	18% (3)	0	6% (1)	12% (2)	22.2
van de Sande et al. [23]	15	Osteoarticular allograft	32	34	228	0	0	23% (3)	8% (1)	15% (2)	—	22.8
Yao et al. [54]	23	Osteoarticular allograft/TBIR	32	19	62	0	77% (10)	38% (5)	8% (1)	23% (3)	38% (5)	—

*N*, patients' number; F-U, follow-up; PSA, pseudoarthrosis (nonunion/severe union delay). ^*∗*^Data referring to the whole population of the study, in which allografts are only a subpopulation.

**Table 2:** Summary data for autograft studies.

Study	Resection (cm)	Graft type	*N*	Age (y)	F-U (m)	Hardware issues	Fractures	Infections	PSA	Failures	MSTS score
Kumar et al. [61]	14	(Arthrodesis) FVFG	4	24	51	4% (1)	0	4% (1)	0	0	—
Mimata et al. [62]	—	(Arthrodesis) FVFG	5	21	75	0	40% (2)	0	0	0	21.4
Bilgin [63]	—	FVFG	9	38	60	22% (2)	11% (1)	11% (1)	0	0	24
Manfrini et al. [64]	13	FVFG	11	5	110	0	64% (7)	9% (1)			22.9
Wada et al. [14]	20	FVFG	8	27	70	0	0	0	12% (1)	12% (1)	23.7
Li et al. [65]	—	Allograft + FVGF	6	16	19	0	0	0	0	0	28
Liu et al. [29]	—	TBIR + FVFG	16	32	62	0	6% (1)	0	25% (4)	25% (4)	19
Amin and Ebeid [66]	14	Scapular pillar autograft	16	21	36	0	0	0	12% (2)	12% (2)	22.5
Padiolleau et al. [67]	12	Scapular pillar autograft	12	36	59	0	0	0	25% (3)	25% (3)	21.3
Barbier et al. [68]	9	CPH autograft	7	8–18	40	14% (1)	29% (2)	0	71% (5)	71% (5)	21.7
Calvert et al. [69]	14	CPH autograft	4	6	43	0	0	0	50% (2)	50% (2)	26-27
Kitagawa et al. [27]	—	CPH autograft	7	29	18	0	0	0	0	0	21.5
Rödl et al. [28]	—	CPH autograft	15	18	59	0	22% (4)	22% (4)	22% (4)	22% (4)	24.6
Tsukushi et al. [70]	—	CPH autograft	7	36	26	14% (1)	0	0	0	0	20.7

*N*, patients' number; F-U, follow-up; PSA, pseudoarthrosis (nonunion/severe union delay); FVFG, free vascularized fibular graft; CPH, clavicula pro humero.

**Table 3:** Summary data for prosthetic studies.

Study	Resection (cm)	Prosthesis type	*N*	Age (y)	F-U (m)	Aseptic loosening	Nerve injury	Instability	Infections	Scapular notching	Reinterventions	Failure	MSTS score	Active ROM
Angelini et al. [43]	11.0	EndoPT (M)	33	46	94	2% (1)	0	15% (8)	4% (2)	—	35% (19)	22% (12)	25	—
Asavamongkolkul et al. [13]	—	EndoPT (CM/M)	33	33	81^*∗*^	3% (2)^*∗*^	0	10% (6)^*∗*^	3% (2)^*∗*^	—	20%^*∗*^	7% (4)^*∗*^	22.8	—
Cannon et al. [100]	14	EndoPT (CM/M)	83	55	30	0	0	6% (5)	2% (2)	—	1% (1)	1% (1)	18.9	Flex42° Abd41°
Fuhrmann et al. [102]	—	EndoPT (M)	21	57	47	0	0	0	5% (1)	—	9% (2)	0%	18	—
Kiss et al. [42]	—	EndoPT (M)	36	42	56^*∗*^	0	6% (2)	25% (9)	3% (1)	—	6% (2)	6% (2)	19.7	—
Liu et al. [29]	11	EndoPT (CM)	25	30	55	36% (9)	0	0	0	—	36% (9)	36% (9)	19.1	—
Manfrini et al. [48]	15.3	EndoPT (M)	25	10	53	12% (3)	0	0	12% (3)	—	24% (6)	16% (4)	20.9	—
Raiss et al. [41]	13	EndoPT (M)	39	60	38	3% (1)	0	10.2% (4)	5% (2)	—	12% (5)	10% (4)	19	Flex34° Abd33°
Rödl et al. [28]	17	EndoPT (M)	19	37	59	0	0	0	0	—	0	0	23.6	—
Potter et al. [30]	13.6	EndoPT (M)	16	54	34	0	0	19% (3)	0	—	25% (4)	25% (4)	20.7	—
van de Sande et al. [23]	9.6	EndoPT (M)	14	45	120^*∗*^	0	0	7% (1)	0	—	7% (1)	7% (1)	23.1	—
Wei et al. [103]	20.6	EndoPT (M)	20	24	40	10% (2)	15% (3)	5% (1)	0	—	5% (1)	5% (1)	21.5	—
Bonnevialle et al. [44]	10.5	Reverse shoulder (P)	10	55	42	0	10% (1)	30% (3)	0	30% (3)	20% (2)	10% (1)	20.2	Flex122°
De Wilde et al. [17]	—	Reverse shoulder (P)	4	42	38	0	0	0	0	0	0	0	27–29	Flex169° Abd172°
De Wilde et al. [104]	—	Reverse shoulder (P)	14	45	92	1	0	2	1	29% (4)	14% (2)	14% (2)	—	Abd157°
Griffiths et al. [45]	—	Reverse shoulder (P)	42	46	71	0	0	26% (14)	5% (2)	0	9% (4)	0%	21.7	—
Guven et al. [46]	10.2	Reverse shoulder (P)	10	49	18	0	0	20% (2)	0	20% (2)	20% (2)	0%	23.4	Flex96° Abd88°
Hu et al. [105]	11.8	Reverse shoulder (P)	7	35	24	0	0	0	0	0	0	0	25.7	Flex133 Abd137
Kaa et al. [49]	11	Reverse shoulder (P)	15	42	46	13% (2)	0	7% (1)	7% (1)	0	27% (4)	20% (3)	23	Flex98° Abd78°
Maclean et al. [106]	9.3	Reverse shoulder (P)	8	49	43	12% (1)	12% (1)	0	0	12% (1)	0%	0%	18	Flex71° Abd62°
Streitbuerger et al. [47]	15.1	Reverse shoulder (P)	18	42	34	0	0	22% (4)	6% (1)	0	6% (1)	6% (1)	25.1	Flex84° Abd80°

*N*, patients' number; F-U, follow-up. ^*∗*^Data referring to the whole population of the study, in which prostheses are only a subpopulation.

**Table 4:** Summary data for APC studies.

Study	Malawer classification	Reconstruction type	Ce.	*N*°	Age (y)	F-U (m)	Aseptic loosening	Fractures	Instability	Infections	PSA	Total failures	MSTS score
Abdeen and Healey [34]	—	Allograft + EndoPT	No	36	—	60	8% (3)	0	3% (1)	0	11% (4)	19% (7)	Lower in extra articular resection
Black et al. [25]	1A	Allograft + EndoPT	Yes	6	41	55	0	0	0	0	17% (1)	33% (2)	69% (21)
El Beaino et al. [130]	1A	Allograft − EndoPT	Yes	21	41	97	14% (3)	5% (1)	57% (12) (1 rev.)	5% (1)	48% (10)	10% at 5 y FU	78% (23)
King et al. [129]	1A	Allograft + RSR	Yes	2	31	51	0	0	0	0	100% (2)	100% (2)	—
Lazerges et al. [131]	1A	Allograft + RSR	Yes	6	66	71	0	0	17% (1)	0	17% (1)	17% (1)	73% (22)
Moran and Stalley [127]	1B	Allograft + EndoPT	No	11	22	70	0	0	36% (4) (3 rev.)	0	18% (2)	54% (6)	66% (20)
Ruggieri et al. [26]	1A	Allograft + resurface	Yes	14	35	25	0	21% (3)	0	7% (1) (rev.)	0	21% (3)	77% (23)
Sanchez-Sotelo et al. [132]	—	Allograft ± RSR	Yes	26	62	48	0	8% (2)	4% (1)	4% (1)	11% (3)	23% (6)	—
Potter et al. [30]	—	Allograft + EndoPT	Yes	16	56	24	0	6% (1)	19% (3)	13% (2)	6% (1)	6% (1)	79% (24)
van de Sande et al. [23]	IA	Allograft + EndoPT	Yes	10	34	204	0	20% (2)	40% (4)	20% (2)	30% (3)	30% (3)	72% (22)

Ce, cement; *N*, patients' number; F-U, follow-up; PSA, pseudoarthrosis (nonunion/severe union delay).
